# Metaplastic changes in the epithelium of radicular cysts: A series of 711 cases

**DOI:** 10.4317/jced.52846

**Published:** 2016-12-01

**Authors:** Igor Tsesis, Eyal Rosen, Liz Dubinsky, Amos Buchner, Marilena Vered

**Affiliations:** 1 Department of Endodontology, Maurice and Gabriela Goldschleger School of Dental Medicine, Tel Aviv University, Tel Aviv, Israel; 2 Private practice; 3 Department of Oral Pathology and Oral Medicine, Maurice and Gabriela Goldschleger School of Dental Medicine, Tel Aviv University, Tel Aviv, Israel

## Abstract

**Background:**

This study was aimed to evaluate the prevalence of metaplastic changes in the epithelium of radicular cysts and to investigate how they relate to the clinical and radiographic characteristics of the cysts, based on a large series of radicular cysts.

**Material and Methods:**

Biopsies of cysts of endodontic origin that were examined at the Department of Oral Pathology between 2004 and 2011 have been re-evaluated for this study. Only cases that were re-confirmed with clinical and histological diagnoses of a radicular or residual radicular cyst were included. The included cases were evaluated for the prevalence of metaplastic changes in the form of mucous secreting cells (MSC) or ciliated cells (CC). The relations between the metaplastic changes and the cyst type (radicular or residual radicular), as well as demographic, clinical and radiographic parameters, were statistically evaluated using Fischer and chi-square tests. Significance was set at *p*<0.05.

**Results:**

A total of 711 cysts were included: 677 were radicular cysts (95%) and 34 (5%) were residual radicular cysts. 23 cases had histopathological diagnoses other than radicular or residual radicular cysts and were excluded from the study. MSC were present in 47 (6.6%) cysts. MSC were significantly more common in residual radicular cysts than in radicular cysts [8 (23.5%) and 39 (5.8%), respectively; *p*<0.001]. MSC-containing cysts were commonly found in asymptomatic patients (10.5%, *p*<0.001), and usually presented with well-defined radiographic borders (7.2%, *p*<0.05). CC were present in 34 (4.8%) cysts, with a markedly high prevalence in the maxillary molar sextant (15%, *p*<0.001).

**Conclusions:**

In the epithelium of radicular and residual radicular cysts the presence of specific metaplastic changes may be related to cyst type, symptomatology, radiographic findings and tooth location.

** Key words:**Radicular cyst, metaplasia, mucous secreting cells, ciliated cells.

## Introduction

Radicular cyst is a sequel of a chronically inflamed granulation tissue located adjacent to the apex of a non-vital tooth with necrotic and infected pulp. It is formed when the epithelial rests of Malassez from the periodontal ligament are stimulated to proliferate to the degree that the central area undergoes necrosis, and a lumen is formed (1-13). In some cases a radicular cyst remains within the jawbones after extraction of the offending tooth. In these cases, the cyst is referred to as a residual radicular cyst (4,7,11,14-16).

Metaplastic changes as the presence of mucous cells or ciliated cells in the epithelial lining of radicular and residual radicular cysts have been reported in the literature in a few studies, all based on a small number of cases (17-22).

Metaplastic changes (metaplasia) are defined as the replacement of one differentiated cell type with another mature differentiated cell type, such as the replacement of squamous epithelium with mucous secreting cells (MSC) or with ciliated cells (CC). The prevalence and possible clinical significance of these metaplastic changes in radicular cysts have not been fully investigated (17-22). Thus, the aim of the study was to evaluate the prevalence of these metaplastic changes in the epithelium of radicular cysts of endodontic origin in a large series of cases, and to investigate how they relate to the clinical and radiographic characteristics of the cysts.

## Material and Methods

Biopsies of cysts of endodontic origin, examined at the Department of Oral Pathology between 2004 and 2011 were collected and re-evaluated. The study was approved by the ethics committee of the institution. The patients’ medical records were retrieved and evaluated for suitability.

Inclusion criteria were comprised of a re-confirmed clinical diagnosis of a peri-radicular pathology of endodontic origin, along with a histological diagnosis of a radicular cyst or residual radicular cyst. Cases with histopathological diagnoses not associated with a cyst of endodontic origin were excluded.

Tissue samples were fixed in 10% buffered formalin and embedded in paraffin. For each case, random 5-micron sections were cut and stained with hematoxylin and eosin. The cases were histologically re-assessed by an experienced oral pathologist (M.V), who evaluated the slides by means of a light microscope (Olympus BH2, Tokyo, Japan).

A diagnosis of a radicular cyst was made for lesions that were clinically associated with non-vital teeth, and microscopically demonstrated a lumen at least partially lined by stratified squamous epithelium and a connective tissue wall with various amounts of chronic and acute inflammatory cells. A diagnosis of residual radicular cyst was made in cases with a reported prior extraction of a non-vital tooth with a peri-radicular lesion, and with microscopic features similar to those of a radicular cyst (3,4,7,8,13-16).

In the cases that fulfilled the inclusion criteria the epithelial lining of the cysts were investigated for the presence of metaplasia in terms of emergence of MSC and CC among the cystic lining epithelial cells. MSC were identified as luminal cells of varying sizes and shapes with a slightly basophilic, granular cytoplasm, appearing either as a continuous row or as scattered cells at irregular intervals. CC were identified as luminal ciliated cells associated with either mucous cells or tall epithelial cells (17,19,21,22).

The medical records of the patients were subjected to data retrieval and analysis. Demographic data included the age and gender of the patients. Clinical data included the type of the involved tooth/teeth, presence of pain or swelling, and whether the patient had a previous root canal treatment. Radiographic data included the location of the cyst, the nature of the cyst borders (well- or poorly-defined) and the presence of border cortication. Whenever the cyst involved more than one region, the location was determined based on the center of the cyst.

The demographic, clinical, radiological and histopathological data were submitted to a statistical analysis. The associations between the presence of MSC / CC and the demographic, clinical and radiographic parameters were analyzed by Fischer and chi-square tests using the SPSS software, version16 (Chicago, IL, USA). The level of significance was set to *p*<0.05.

## .Results

A total of 734 biopsies of cysts of endodontic origin were submitted to re-evaluation. Of these, 23 cases were excluded from the study: 3 cases were diagnosed as keratocystic odontogenic tumor, and 20 cases were devoid of epithelial lining, which was probably destroyed by inflammatory infiltrate. Eventually 711 cases were evaluated, including 677 (95%) radicular cysts and 34 (5%) residual radicular cysts.

Age was reported in the records of 583 patients, and ranged from 4 to 97 years, with a mean of 42±15 yrs. Gender was reported in the records of 703 patients, with 358 (50.9%) females and 345 (49.1%) males.

**Figure 1:**
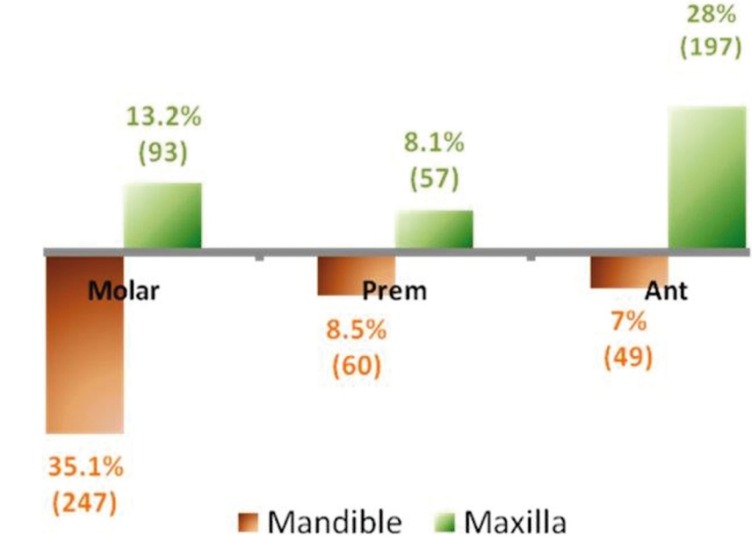
Distribution of the radicular cysts by sextants (% was calculated from the total number of cysts, N=711).“Prem” - pre-molars, “Ant” - anterior teeth.

The location of the cyst was known in 703 of the cases. Most of the cysts were located in the mandibular molar (35.1%) and maxillary anterior (28%) regions (Fig. 1).

Data on clinical symptoms (pain or swelling) was available for 443 patients: 224 (50.6%) were asymptomatic while 219 (49.4%) were symptomatic.

A previous root canal treatment was present in 307 (43.2%) teeth.

The appearance of radiographic borders was determined for 379 cysts: 333 (87.9%) were well-defined while 46 (12.1%) were ill-defined. The presence of border cortication was known in 251 cysts, with 158 (62.9%) being uncorticated and 93 (37.1%) corticated.

**Figure 2:**
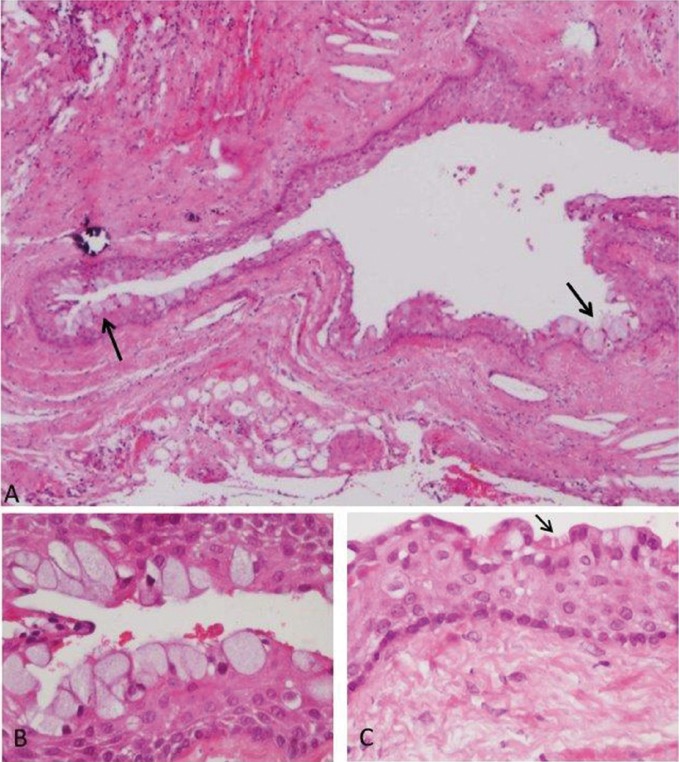
A) Fragment of a radicular cyst wall that shows metaplastic changes of mucous secreting cells (arrows; hematoxylin and eosin, x100 original magnification). B) Magnification of the mucous cells. C) Ciliated cells (arrow).

A total of 66 (9.3%) cysts showed metaplastic changes: MSC were present in 47 (6.6%) and CC in 34 (4.8%) of all cases (Fig. 2 presents a fragment of a radicular cyst lining epithelium that shows metaplastic changes). Out of the 47 cysts with MSC,15 (32%) were accompanied by CC (*p*<0.001).

MSC were significantly more common in residual radicular cysts than in radicular cysts [8 (23.5%)and 39 (5.8%), respectively; *p*<0.001). MSC-containing cysts were distributed among all sextants ( Table 1). MSC were more common in males than in females [33 (9.6%) and 13(3.6%), respectively; *p*=0.002)] and were more common in the asymptomatic than in the symptomatic cysts [23 (10.5%) and 4 (1.8%), respectively; *p*<0.001)]. MSC were found in 24 (7.2%) of the cases with well-defined radiographic borders, and in none of the cases with ill-defined borders (*p*<0.05). There was no association between the presence of MSC and the patients’ age, location of the cysts, previous root canal treatment or radiographic border cortication (*p*>0.05).

**Table 1:**
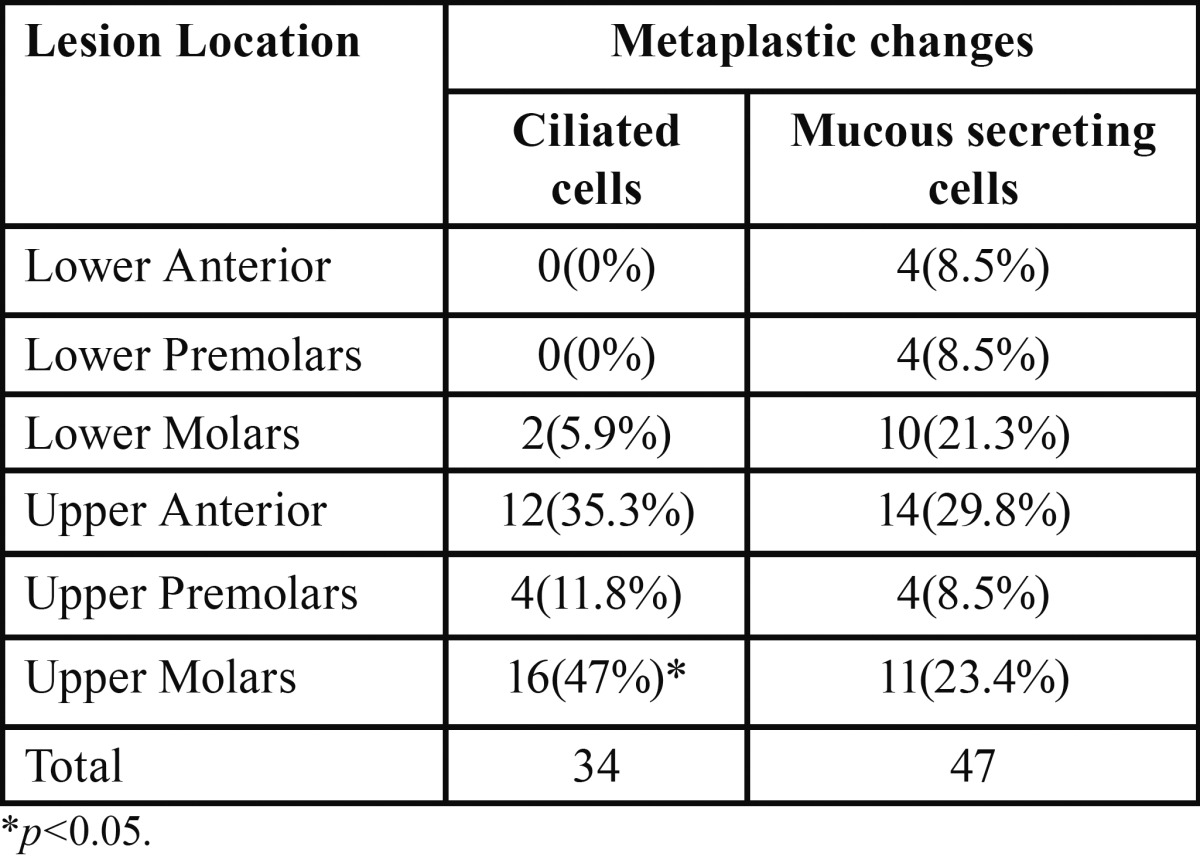
Metaplastic changes in the different cyst locations.

Metaplastic changes of the CC type were found in 33 (4.9%) of the radicular cysts and in 1 (2.9%) of the residual radicular cysts. CC-containing cysts were more common in the upper molar sextant (16 (47%)) than in any other location (*p*<0.001) ( Table 1). There was no statistically significant association between the presence of CC and any other investigated parameter.

## Discussion

In the present study a large series of 711 radicular cysts was evaluated. The findings highlighted the prevalence of MSC (6.6%) in these cysts, and the significant association of MSC with residual radicular cysts, with lack of symptoms, with male gender, and with well-defined radiographic borders. The study also highlighted the prevalence of CC (4.8%) and their significant association with the upper molar teeth, as well as the significant coexistence of MSC and CC together.

In previous studies, MSC were reported in 10-40% of the radicular and residual radicular cysts, mostly in the outer layers of the epithelium, sometimes as single cells and sometimes forming a gland-like structure (17,21,22). MSC were reported as more pre-valent in cystic lesions of the maxilla, in which their numbers increased with the cyst age (about 7% every decade) (17,22). CC were reported in 0.7-11% of radicular cysts, usually in the luminal layer of the epithelium, covering only small fractions of the cyst lumen and they were usually accompanied by the presence of MSC (17,22). As CC were reported to be more prevalent in maxillary cysts, it was suggested that they migrated from the maxillary sinuses mucosa (17,19,21). However, since MSC and CC have also been found in the mandible, it seems that their presence in the cyst epithelium is a result of local metaplasia rather than cell migration (17,22).

MSC prevalence of 6.6% found in the present series of radicular cysts is lower than the range of 10% to 40% reported in previous studies, but CC prevalence of 4.8% falls within the previously reported range of 0.7% to 10.7% (14,17,20-22). While in this study it was found that MSC occurred mainly in residual radicular cysts, others previously reported the presence of MSC mainly in radicular cysts (14). It should be noted that not only did previous studies include a lower number of cases (range of 33 to 402 cases, compared to 711 in the present study), they were also heterogeneous in the types of peri-radicular lesions examined- some included radicular cysts only, others residual radicular cysts only, and some included both types of lesions under the term “dental cysts” without specifying their sub-classification (14,17,20-22).

We found no association between the presence of MSC and tooth location, similar to previously reported data (17,18). In contrast, CC were significantly associated with the maxillary molars, similar to the finding of Takeda *et al.* 2005 (22), who reported a high prevalence of CC in the maxilla. It should be noted that in the present study, CC were also found in cysts related to mandibular molars, albeit in a small number of cases. It can therefore be inferred that CC should not always be regarded as an extension of the maxillary sinus lining but rather as a true metaplastic change (21).

Tosh *et al.* (23) have suggested that the metaplastic changes are not an outcome of phenotypic changes within a mature, differentiated cell, but rather a result of a reprogramming process, normally occurring in precursor cells within any tissue (23). It may be assumed that precursor cells do reside among the epithelial rests of Malassez and that they convey this type of epithelium a certain degree of plasticity. The reprogramming process is mediated by cytokine-derived signals, various growth factors, as well as by modifications in the extra-cellular matrix characteristics and specific genes involved in the metaplastic process are being revealed (23).

It remains to be investigated whether the presence of metaplastic changes (MSC, CC) in radicular cysts has a clinical impact either on the potential of these cysts to heal after a root canal treatment or on the persistence of the residual radicular cysts after tooth extraction that removes the irritant factors. Alternatively, it is possible that residual radicular cysts persist after tooth extraction because these cysts are not curetted at the time of extraction, and the resulting conditions facilitate metaplastic changes (i.e. the metaplasia may be a result of the residual cysts, it may be a factor influencing their outcomes, or both).

It may be concluded that in the epithelium of radicular cysts, the presence of metaplastic changes may be related to cyst type, symptomatology, radiographic findings, and tooth location. The influence of metaplastic cells in cysts of endodontic origin on the prognosis of endodontic treatment remains to be further investigated.
